# Co-existence of Age-Related Macular Degeneration and Diabetic Retinopathy in a Tertiary Referral Center in Greece

**DOI:** 10.7759/cureus.31051

**Published:** 2022-11-03

**Authors:** Evgenia Bourouki, Eleni Dimitriou, Athanasios Chatzipantelis, Petros Kapsis, Georgios Theodossiadis, Panagiotis Theodossiadis, Irini Chatziralli

**Affiliations:** 1 Ophthalmology, National and Kapodistrian University of Athens, Athens, GRC; 2 Medicine, University of Athens, Athens, GRC; 3 Ophthalmology, Attikon University Hospital, Athens, GRC

**Keywords:** diabetic retinopathy, retina, fundoscopy, age-related macular degeneration, epidemiology

## Abstract

Purpose: To investigate the co-existence of diabetic retinopathy (DR) and age-related macular degeneration (AMD), based on five-year data in a University setting.

Methods: Participants in the study included 1739 patients with diabetes mellitus, who were examined in our setting from 2015 to 2019. The presence of DR and AMD was recorded while the clinical characteristics of patients were evaluated.

Results: In our study sample, 183 out of 1739 patients with diabetes mellitus (10.5%) were diagnosed with AMD, 116 without any sign of DR, and 67 with DR. In the group of patients with DR, dry AMD was noticed mostly in patients with mild non-proliferative DR (NPDR) (11.5% dry AMD) compared to those with moderate NPDR (4.5% dry AMD), severe NPDR (4.2%) and proliferative DR (PDR) (2.4%). Similar results were found for neovascular AMD (3% in mild NPDR, 1.9% in moderate NPDR, 1% in severe NPDR, and 1.8% in PDR). There was a significant correlation between the co-existence of both diseases and the severity of DR, with AMD being less prevalent in patients with more severe DR. In patients with diabetic macular edema, dry AMD was observed in 12 (4.6%) and neovascular AMD in nine (3.4%).

Conclusions: The five-year prevalence of AMD in DR patients was 9% while in diabetic patients without DR it was found to be 11.5%. Therefore, the co-existence of DR and AMD is not common, suggesting that DR may be protective for AMD development.

## Introduction

Diabetes mellitus is a major worldwide health concern with a significantly increased tendency [1]. Diabetic retinopathy (DR) is one of its serious microvascular complications and has been reported to be one of the major causes of blindness, especially among adults younger than 40 years in the developed world [2,3]. In particular, it is estimated that about 400 million people are affected by diabetes mellitus with one-third of them having DR and one-tenth having vision-threatening disease course, including diabetic macular edema (DME) and proliferative DR (PDR) [1,4].

Age-related macular degeneration (AMD) is the most common cause of legal blindness in the elderly, especially among Caucasians in the developed world [5]. The pathophysiology of AMD is multifactorial and not fully known, although several risk factors have been identified [6-9]. Age is considered the most important demographic risk factor while smoking and sunlight exposure are aggravating environmental factors. Nutritional factors, gut microbiota, hypertension, and genetic variations have also been reported to be risk factors for AMD [6-9].

Although several studies have suggested an association between AMD and diabetes mellitus, mainly because of their common pathophysiology background involving inflammatory processes and oxidative stress, there is a controversy regarding the association between the two clinical entities [10-14]. According to the Beaver Dam Eye Study, diabetes mellitus is not related to early AMD or geographic atrophy, although male patients older than 75 years, with diabetes, had a higher frequency of exudative AMD than those without diabetes [10]. The European Eye (EUREYE) Study investigated the association between diabetes mellitus and AMD severity and found a positive correlation between a history of diabetes and neovascular AMD, but not with geographic atrophy or early AMD [11]. Furthermore, the Blue Mountains Eye Study reported that diabetes mellitus was significantly associated with the prevalence of geographic atrophy, but no association was found for either neovascular AMD or early AMD [12]. On the contrary, other studies failed to find any association between diabetes mellitus or DR and AMD; maybe because diabetic patients may not live long enough to develop AMD [13,14].

It is also worth noting that previous studies indicated a significantly lower prevalence of AMD among patients with diabetes mellitus than in the general population [15]. Of note, the correlation between AMD and DR was poorly documented. Benson et al. highlighted the uncommon presence of AMD in patients with DR [16] while Zylbermann et al. suggested that the prevalence of AMD among patients with DR was very low and that the patients with DR treated by laser photocoagulation were found to be less prone to develop neovascular AMD [17]. Based on the above, the purpose of the present study was to investigate the association between DR and AMD, based on five-year data in a University setting.

## Materials and methods

In this study, there were 1739 patients with diabetes mellitus who underwent screening in the joint diabetology/ophthalmology department of the Attikon University Hospital in Athens, Greece between 1st January 1 2015 and 31st December 2019. Patients with dense cataracts or media opacities, which preclude a reliable fundoscopy, were excluded. The study was conducted as per the tenets of the Declaration of Helsinki and was approved by the Institutional Review Board of Attikon University Hospital (approval no.: 698/2019). Informed consent was obtained from all participants before entering the study.

The demographic characteristics (age and gender), medical history, and comorbidities (hypertension, hyperlipidemia) of all participants were recorded. All patients underwent a standard ophthalmic examination including best-corrected visual acuity (BCVA) measurement, intraocular pressure (IOP) measurement, slit-lamp examination, and dilated fundoscopy while optical coherence tomography (OCT) was also performed using SPECTRALIS® HRA+OCT (Heidelberg, Tampa, FL, USA). Specifically, for each patient, six radial scans, 3 mm long, were performed at equally spaced angular orientations centered on the foveola. Fluorescein angiography was done at the physician’s discretion, if needed, to confirm the diagnosis.

The stage of the DR was determined by dilated fundoscopy, and patients were classified according to the severity of DR as follows: no apparent DR, mild non-proliferative DR (NPDR), moderate NPDR, severe NPDR and proliferative DR (PDR) [18]. The presence of diabetic macular edema was also recorded. All participants were also evaluated for the presence of abnormalities related to AMD, either dry or neovascular. The diagnosis of dry AMD has been characterized by the presence of retinal pigment epithelium (RPE) changes, Bruch membrane alterations, and drusen while neovascular AMD has been diagnosed when choroidal neovascularization is detected.

Statistical analysis was performed using the Statistical Package for Social Sciences (SPSS) version 22.0 (IBM Corp., Armonk, NY, USA). Descriptive statistics, including the mean values, median, standard deviations, and percentages, were used to describe the patients’ characteristics. Correlations were calculated with Spearman’s rank correlation coefficient. Statistical significance was set to p<0.05.

## Results

Table 1 shows the demographic and clinical characteristics of our study sample. The mean age of patients was 68.9±8.2 years. Of the 1739 patients, 742 were male (42.7%) and 997 were female (57.3%). In our study sample, 1393 patients were diagnosed with type 2 diabetes mellitus (80.1%) and 346 with type 1 (19.9%). As far as DR staging is concerned, 1006 out of 1739 patients with diabetes showed no signs of DR (57.9%), 315 patients had mild NPDR (18.1%), 157 patients had moderate NPDR (9%), 96 patients had severe NPDR (5.5%), and 165 patients had PDR (9.5%) while 263 subjects (15.1%) presented with diabetic macular edema at the time of the initial examination.

**Table 1:** Demographic and clinical characteristics of our study sample

Age (mean ± SD, years)	68.9±8.2
Gender (n, %)	
Male	742 (42.7%)
Female	997 (57.3%)
Type of diabetes mellitus (n, %)	
Type 1	346 (19.9%)
Type 2	1393 (80.1%)
Diabetic retinopathy severity (n, %)	
No diabetic retinopathy	1006 (57.9%)
Mild non-proliferative diabetic retinopathy	315 (18.1%)
Moderate non-proliferative diabetic retinopathy	157 (9.0%)
Severe non-proliferative diabetic retinopathy	96 (5.5%)
Proliferative diabetic retinopathy	165 (9.5%)

**Figure 1:**
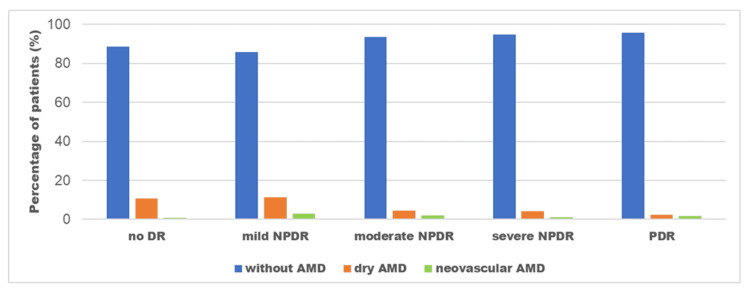
Percentage of patients with AMD (dry or neovascular) at different stages of DR, namely mild NPDR, moderate NPDR, severe NPDR, and PDR. AMD: Age-related macular degeneration, DR: Diabetic retinopathy, NPDR: Non-proliferative diabetic retinopathy, PDR: Proliferative diabetic retinopathy

Regarding the presence of AMD, 183 out of 1739 patients with diabetes (10.5%) were diagnosed with AMD. In particular, 116 out of 1006 patients without any sign of DR (11.5%) exhibited AMD (108 dry AMD and eight neovascular AMD). In the group of patients with DR, AMD was observed in 67 out of 733 patients (9.1%). Figure 1 shows the exact percentage of patients with AMD based on the DR stage. More specifically, 11.5% of patients with mild NPDR had dry AMD and 3% had wet AMD. Of the 157 patients with moderate NPDR, seven had dry AMD (4.5%) and three had neovascular AMD (1.9%). Of the 96 patients in the severe NPDR group, four patients had dry AMD (4.2%) and only one patient had wet AMD (1%). Finally, four out of 165 patients with PDR developed dry AMD (2.4%), and three other patients developed neovascular AMD (1.8%). There was a statistically significant association between the severity of DR and the presence of AMD (p=0.031 within groups of DR) with patients having more severe DR exhibiting less frequent AMD.

Additionally, out of 263 patients with DME, 242 did not present AMD (92%), whereas 12 patients had dry (4.6%) and nine patients had neovascular AMD (3.4%).

## Discussion

Age-related macular degeneration and DR are among the leading causes of vision loss in the world [2,5]. The principal message of our study was that the co-existence of DR and AMD is not usual. Specifically, in our study, the five-year prevalence of AMD in DR patients was 9% while in diabetic patients without DR it was found to be 11.5%. Another finding of our study was the correlation between the co-existence of both diseases and the severity of DR since the prevalence of AMD in patients with severe DR and PDR is significantly lower compared to patients with mild NPDR or no DR. Our findings were per the majority of recent studies, reporting that the prevalence of AMD is lower in diabetic patients than in the general population [15]. However, other studies suggested that DR, both NPDR, and PDR may predispose individuals to an increased risk of AMD [19].

To explain the low prevalence of AMD in patients with DR, the underlying pathogenetic mechanisms of both diseases have to be taken into account. The earliest signs of AMD are drusen i.e., debris in Bruch’s membrane, and their formation is affected by choriocapillaris’ dysfunction due to low metabolic activity of the outer retina and retinal pigment epithelium (RPE) [20]. It is well known that both choroidal blood volume and flow are reduced with aging increasing the risk of AMD [20]. In addition, the choriocapillaris lose their ability to supply RPE cells with oxygen and maintain the outer retina homeostasis. Consequently, the dysfunction or death of RPE cells results in alterations of vascular endothelial growth factor (VEGF) secretion. When the microenvironment of the choriocapillaris experiences a VEGF deficit, one possible response can be extensive atrophy, as shown in dry AMD. In the case of neovascular AMD, the hypoxic stress on both retina and RPE cells leads to VEGF overproduction, which results in abnormal neovascularization [20]. On the other hand, DR is mainly a microangiopathy, characterized by progressive thickening of the basement membrane of the retinal capillary wall, loss of pericytes, proliferation of endothelial cells, and drop-out of the choriocapillaris [4]. Diabetic retinopathy is essentially an inner retina disease while AMD affects mainly the outer retina. It is well known that the VEGF concentration in the vitreous of eyes with DR progressively increases depending on the severity of DR. The high concentration of diffusible VEGF can be sufficient for the survival of RPE, acting as a protective factor and at the same time maintaining the homeostasis of the choriocapillaris, diminishing also the deposition of debris that forms drusen [13]. Saravia et al. proposed this mechanism as a possible explanation of how the presence of DR can prevent the degeneration of a still-functioning RPE, supplying it with sufficient oxygenation [13].

According to Zylbermann et al., it seems that the more advanced the DR, the more pronounced the prevention of AMD is [17]. Perhaps the therapeutic approach of DR plays a significant role to slow the progression of AMD. Patients with severe DR or PDR who have been treated by laser photocoagulation are less likely to develop AMD [17]. Yoshikawa et al. highlighted the beneficial effect of laser photocoagulation in halting the progression of AMD, supporting the view that patients with DR treated with laser photocoagulation are less likely to develop AMD [21].

Furthermore, in our study, patients with DME had a lower prevalence of dry and neovascular AMD. Our observations were in line with the previous studies, which could not find any correlation between diabetic retinal pigment epitheliopathy or DME and AMD [22]. It is worth noting that screening patients with diabetes not only for DR but also for the presence of AMD using retinal fundus photographs is reliable and cost-effective [22].

A potential limitation of our study is that it was based on a specialized department of a tertiary center (DR clinic) and was not population-based, implying potential selection bias. Another bias of the study could be the mean age of the study sample. The selective mortality, which is increased in patients with diabetes mellitus, may result in a selectively diminished prevalence of AMD in patients with diabetes mellitus and DR since the elderly population is most affected by AMD. However, this study is based on five-year data and no similar studies have been performed in our country to provide epidemiological data on the co-existence of DR and AMD.

## Conclusions

In conclusion, we found that the five-year prevalence of AMD in DR patients was 9% while in diabetic patients without DR it was found to be 11.5%. Moreover, there was a significant correlation between the co-existence of both diseases and the severity of DR, with AMD being less prevalent in patients with more severe DR. The clinical relevance of our results suggests that possible pathogenetic underlying mechanisms could act as protective factors preventing the development of AMD in patients with DR. Further population-based studies with larger sample sizes are needed to justify our results.

## References

[1] International Diabetes Federation (2022). Resources | IDF Diabetes Atlas. Brussels, Belgium: International Diabetes Federation.

[2] Das A (2016). Diabetic retinopathy: battling the global epidemic. Invest Ophthalmol Vis Sci.

[3] Congdon NG, Friedman DS, Lietman T (2003). Important causes of visual impairment in the world today. JAMA.

[4] Ting DS, Cheung GC, Wong TY (2016). Diabetic retinopathy: global prevalence, major risk factors, screening practices and public health challenges: a review. Clin Exp Ophthalmol.

[5] Pascolini D, Mariotti SP, Pokharel GP, Pararajasegaram R, Etya'ale D, Négrel AD, Resnikoff S (2004). 2002 global update of available data on visual impairment: a compilation of population-based prevalence studies. Ophthalmic Epidemiol.

[6] Tomany SC, Wang JJ, Van Leeuwen R (2004). Risk factors for incident age-related macular degeneration: pooled findings from 3 continents. Ophthalmology.

[7] Heesterbeek TJ, Lorés-Motta L, Hoyng CB, Lechanteur YT, den Hollander AI (2020). Risk factors for progression of age-related macular degeneration. Ophthalmic Physiol Opt.

[8] Zhang QY, Tie LJ, Wu SS (2016). Overweight, obesity, and risk of age-related macular degeneration. Invest Ophthalmol Vis Sci.

[9] Zisimopoulos A, Klavdianou O, Theodossiadis P, Chatziralli I (2021). The role of the microbiome in age-related macular degeneration: a review of the literature. Ophthalmologica.

[10] Klein R, Klein BE, Moss SE (1992). Diabetes, hyperglycemia, and age-related maculopathy. The Beaver Dam Eye Study. Ophthalmology.

[11] Topouzis F, Anastasopoulos E, Augood C (2009). Association of diabetes with age-related macular degeneration in the EUREYE study. Br J Ophthalmol.

[12] Mitchell P, Wang JJ (1999). Diabetes, fasting blood glucose and age-related maculopathy: The Blue Mountains Eye Study. Aust N Z J Ophthalmol.

[13] Saravia M, Zeman L, Ingolotti M, Schlaen A (2017). The VEGF paradox: Does diabetic retinopathy protect from age related macular degeneration?. Med Hypotheses.

[14] Hyman LG, Lilienfeld AM, Ferris FL 3rd, Fine SL (1983). Senile macular degeneration: a case-control study. Am J Epidemiol.

[15] Borrone R, Saravia M, Bar D (2008). Age related maculopathy and diabetes. Eur J Ophthalmol.

[16] Benson WH, Farber ME (1989). Diabetic retinopathy in a rural diabetic population. Prevalence and risk. W V Med J.

[17] Zylbermann R, Landau D, Rozenman Y, Abrahami S, Pollack A (1997). Exudative age-related macular degeneration in patients with diabetic retinopathy and its relation to retinal laser photocoagulation. Eye (Lond).

[18] Wilkinson CP, Ferris FL 3rd, Klein RE (2003). Proposed international clinical diabetic retinopathy and diabetic macular edema disease severity scales. Ophthalmology.

[19] Hahn P, Acquah K, Cousins SW, Lee PP, Sloan FA (2013). Ten-year incidence of age-related macular degeneration according to diabetic retinopathy classification among medicare beneficiaries. Retina.

[20] Ramrattan RS, van der Schaft TL, Mooy CM, de Bruijn WC, Mulder PG, de Jong PT (1994). Morphometric analysis of Bruch's membrane, the choriocapillaris, and the choroid in aging. Invest Ophthalmol Vis Sci.

[21] Yoshikawa T, Ogata N, Wada M, Otsuji T, Takahashi K (2011). Characteristics of age-related macular degeneration in patients with diabetic retinopathy. Jpn J Ophthalmol.

[22] Gangwani R, Lai WW, Sum R, McGhee SM, Chan CW, Hedley AJ, Wong D (2014). The incidental findings of age-related macular degeneration during diabetic retinopathy screening. Graefes Arch Clin Exp Ophthalmol.

